# Immune risk phenotype is associated with nosocomial lung infections in elderly in-patients

**DOI:** 10.1186/1742-4933-8-8

**Published:** 2011-10-01

**Authors:** A Plonquet, S Bastuji-Garin, F Tahmasebi, C Brisacier, K Ledudal, JP Farcet, E Paillaud

**Affiliations:** 1 Assistance Publique-Hôpitaux de Paris (APHP), hôpital Henri-Mondor, Department of Immunology, Creteil, F 94010, France; 2 Assistance Publique-Hôpitaux de Paris (APHP), hôpital Henri-Mondor, Pôle Recherche Clinique et Santé Publique, Creteil, F 94010 France; 3 Université Paris Est, Faculté de Médecine, LIC EA 4393, Créteil, F 94010, France; 4 Assistance Publique-Hôpitaux de Paris (APHP), hôpital Henri-Mondor, Unité de Recherche Clinique, Creteil, F 94010 France; 5 Assistance Publique-Hôpitaux de Paris (APHP), hôpital Henri-Mondor, Département de Médecine Interne et Gériatrie, Creteil, F 94010 France; 6 Assistance Publique-Hôpitaux de Paris (APHP), hôpital Henri-Mondor, INSERM, Centre d'Investigation Clinique 006, Creteil, F 94010 France

**Keywords:** Immune Risk Phenotype, CMV status, nosocomial infection, elderly patients, immunosenescence

## Abstract

**Background:**

Nosocomial infections are extremely common in the elderly and may be related to ageing of the immune system. The Immune Risk Phenotype (IRP), which predicts shorter survival in elderly patients, has not been evaluated as a possible risk factor for nosocomial infection. Our aim was to assess the prevalence of nosocomial infections in elderly in-patients and to investigate potential relationships between nosocomial infections and the immunophenotype, including IRP parameters.

**Results:**

We included 252 consecutive in-patients aged 70 years or over (mean age, 85 ± 6.2 years), between 2006 and 2008. Among them, 97 experienced nosocomial infections, yielding a prevalence rate of 38.5% (95% confidence interval, 32.5-44.5). The main infection sites were the respiratory tract (21%) and urinary tract (17.1%) When we compared immunological parameters including cell counts determined by flow cytometry in the groups with and without nosocomial infections, we found that the group with nosocomial infections had significantly lower values for the CD4/CD8 ratio and naive CD8 and CD4 T-cell counts and higher counts of memory CD8 T-cells with a significant increase in CD28-negative CD8-T cells. Neither cytomegalovirus status (positive in 193/246 patients) nor presence of the IRP was associated with nosocomial infections. However, nosocomial pneumonia was significantly more common among IRP-positive patients than IRP-negative patients (17/60 versus 28/180; *p *= 0.036).

**Conclusion:**

Immunological parameters that are easy to determine in everyday practice and known to be associated with immune system ageing and shorter survival in the elderly are also associated with an elevated risk of nosocomial pneumonia in the relatively short term.

## Background

Nosocomial infections (NIs) constitute a major public health concern, as they are common and associated with both high morbidity and mortality rates and high healthcare costs [1,2]. Risk factors for NIs vary with the infection site, healthcare setting, and patient age. The National Nosocomial Infections Survey done in the US in 1986-1990 showed that 54% of all NIs occurred in people aged 65 or over [3]. In elderly individuals, the increased susceptibility to severe infections and decreased efficacy of vaccination may reflect ageing of the immune system, or immunosenescence, which involves nearly all the components of the immune system [4-6].

Among the changes characteristic of immunosenescence, those believed to play a major role include the progressive decline in naive circulating T-cell counts (paralleling involution of the thymus), expansion of memory T-cells, and accumulation of terminally differentiated effector CD8 T-cells characterised by loss of CD28 expression [7]. Peripheral CD28^- ^CD8 T-cell counts are elevated in elderly individuals [8-10]. The expansion of this T-cell subset is related to the clonal T-cell expansions often detected in elderly individuals and leads to competition for CD8 T-cell survival niches, thereby hampering the immune responses to infections, most notably those of viral origin [10].

Cytomegalovirus (CMV) is considered crucial among the repeated antigenic stimuli responsible for the accumulation of oligoclonal effector CD8 T cells [11,12]. Actively keeping CMV in its latent state via the anti-viral immune response places an enormous burden on the entire adaptive immune system [13] and makes a substantial contribution to the phenotype changes observed in T-cell subsets in elderly people [14].

Geriatric studies have identified several alterations in immunological parameters that are associated with shorter survival in elderly individuals. The term "immune risk phenotype" (IRP) was defined by Pawelec et al. [15] as the combination of high CD8 and low CD4 T-cell counts with a poor proliferative response to concanavalin A and low percentages of B-cells. The IRP predicted a 2- to 4-year decrease in survival among free-living healthy Swedes over 85 years of age (OCTO study, [16]). Subsequently, the definition of the IRP was simplified to inversion of the CD4/CD8 T-cell ratio [17]. Serological evidence of persistent CMV infection was then found to be strongly associated with CD28^- ^CD8 T-cell expansion and was consequently added to the definition of the IRP [18]. In the NONA longitudinal study of IRP parameters in a cohort of elderly out-patients not selected for good health, the IRP was associated with a higher mortality rate [19]. To the best of our knowledge, immunosenescence as a possible risk factor for NIs in elderly patients has not been evaluated previously.

The aims of this study were to prospectively assess the prevalence of NIs in elderly in-patients and to look for potential relationships between the occurrence of NIs and the immunophenotype (i.e., main lymphocyte subsets and naive and memory/effector T-cells), with special attention to the IRP as defined in the NONA study [19].

## Results

We included 252 patients aged 70 years or over (181 [71.8%] females; mean age, 85.2 [± 6.2]) years). These patients were transferred from acute medical units (n = 224) or orthopaedic surgical units (n = 28) to the geriatric rehabilitation unit of our internal medicine department. Table 1 reports the main clinical characteristics at admission. The most common diagnoses were cardiopulmonary disease (25.8%), dementia (24.2%), and osteoporosis or fracture (20.2%).

**Table 1:** Clinical characteristics of the study population at admission to the geriatric rehabilitation department

	Patients (n = 252)
**Age**, mean ± SD, years	85.2 ± 6.2

**Sex **(male/female)	71/181

**Marital status**, n (%)	
- married	78 (31)
- single	14 (5)
- divorced	4 (2)
- widowed	156 (62)

**Living arrangements**, n (%)	
-home	243 (96)
-nursing home	9 (4)

**Main reason for hospital admission**, n (%)	
- cardiopulmonary disease	65 (25.8)
- dementia	61 (24.2)
- psychiatric disease	14 (5.5)
- stroke	26 (10.3)
- neurological disease	17 (6.7)
- metabolic and other diseases	18 (7.1)
- osteoporosis and/or fracture	51 (20.2)

**Patients with at least one nosocomial infection**, n (%)	97 (38.5)
Sites (n = 115)	
- Respiratory tract, n (%)	53 (21)
*- pneumonia*	*45 (17.9)*
*- bronchitis*	*9 (3.6)*
- Urinary tract, n (%)	43 (17.1)
- Gastrointestinal tract	3 (1.2)
- Skin	11 (4.4)
- Other	5 (2.0)

We recorded 115 infections in 97 patients; 18 patients each had two infections at different sites.

At least one NI was diagnosed in 97 patients, yielding a prevalence rate of 38.5% (95%CI, 32.5-44.5%); 18 patients each had two NIs; thus the total number of NIs was 115. The most common sites of NI were the respiratory tract (21%) and urinary tract (17.1%).

Table 2 compares the groups with and without NIs (NI+ and NI-, respectively). Age and sex did not differ significantly between the groups. The CD4/CD8 ratio was significantly lower in the NI+ group than in the NI- group (*p *= 0.027). Accordingly, although CD4 T-cell counts did not differ significantly between the groups, the absolute CD8 T-cell count tended to be higher in the NI+ group. Naive CD4 T-cells were significantly decreased in the NI+ group when expressed as a percentage, but the difference was no longer significant when the counts were expressed as absolute values. The NI+ group had significantly lower naive CD8 T-cell counts expressed as both the absolute value and the percentage, compared to the NI- group. Counts of peripheral memory CD8 T-cells and CD28^-^CD8 T-cells were significantly higher in the NI+ group, both as absolute values and as percentages. However, in both groups, the median values of all parameters were within the normal age-specific range.

**Table 2:** Immunological characteristics of elderly patients according to occurrence of at least one nosocomial infection within 3 months after admission.

	Total (252 patients)	Nosocomial infection (97 patients)	No nosocomial infection (155 patients)	*p *value
Age in years, mean (SD)	85 ± 6	85 ± 5.4	84 ± 6.2	0.295

Female gender, n (%)	181(67.9)	68 (70.1)	113(72.9)	0.631

CD4 T-cell count*	682 (555-967)	685 (534-951)	680 (561-990)	0.83

CD8 T-cell count*	312 (206-447)	348(207-557)	272 (206-412)	0.06

CD4/CD8 ratio	2.5 (1.4-3.5)	2.1 (1.3-3.2)	2.6 (1. 6-3.8)	0.027

NK cell count*	176 (112-258)	186 (112-262)	160 (110-255)	0.38

B cell count*	126 (78-175)	133 (78-175)	117 (78-184)	0.83

**CD4 T cells**				
**Naive **(CD45RA+CD62L+)%**	17.7 (13.2-22.9)	16.3 (8.8-24.2)	18.7 (12.5-28.1)	0.03
*Absolute value/μL*	*242 (148-388)*	*214 (125-385)*	*260 (155-390)*	0.09
**Memory**				
Peripheral (CD45RA-CD62L-)%**	6.5 (4.5-9.3)	6.6 (4.5-9.5)	6.5 (4.5-9.3)	0.49
*Absolute value/μL*	*99 (56-152)*	*109 (64-156)*	*96 (53-150)*	0.29
Central (CD45RA-CD62L+)%**	18.2 (10.5-26.9	16.9 (12.9-23.1)	18.3 (13.5-22.9)	0.35
*Absolute value/μL*	*256 (175-351)*	*249 (174-358)*	*262 (172-350)*	0.91
CD45RA+CD62L-%**	0.6 (0.3-1.4)	0.6 (0.2-1.4)	0.6 (0.3-1.3)	0.97
*Absolute value/μL*	*9 (3-22)*	*9 (3-24)*	*9 (4-20)*	*0.93*

**CD8 T cells**				
**Naive **(CD45RA+CD62L+)*%*	5 (3.3-7.4)	4.3 (2.9-6.9)	5.2 (3.8-8)	0.006
*Absolute value/μL*	*73 (44-115)*	*67 (39-107)*	*80 (46-125)*	*0.03*
**Memory**				
Peripheral (CD45RA-CD62L-)*%*	2.8 (1.5-4.9)	3.1 (1.6-6.2)	2.6 (1.4-4.0)	0.02
*Absolute value/μL*	*44 (20-76)*	*49 (23-92)*	*37 (17-71)*	*0.01*
Central (CD45RA-CD62L+)*%*	2.7 (1.7-3.9)	3 (1.8-3.9)	2.6 (1.6-4.1)	0.32
*Absolute value/μL*	*38 (23-59)*	*41 (26-61)*	*36 (23-57)*	*0.22*
**Terminal Effector**				
CD45RA+CD62L-*%*	7.9 (4-12.9)	8.5(3.8-16.2)	7.2 (4.1-11.9)	0.15
*Absolute value/μL*	*109 (54-191)*	*135(60-233)*	*101(54-169)*	*0.11*
CD28-*%*	56.7 (42.5-73)	51.8 (40-66)	53.6 (40-68)	0.02
*Absolute value/μL*	*153 (88-272)*	*196 (91-357)*	*139 (88-248)*	*0.01*

IRP criteria***(n = 240)				
CD4/CD8 < 1 (n = 245)	20 (8.2)	10 (10.5)	10 (6.7)	0.34
CD8 T-cells > 600 (n = 245)	32 (13.1)	17 (17.9)	15 (10.0)	0.07
CD28-CD8+ T-cells > 300 (n = 238)	64 (26.9)	31 (33.3)	33 (22.8)	0.07
Positive CMV serology (n = 246)	193 (78.5)	76 (79)	117 (78)	0.87
Positive IRP (n = 240, 95/145)	60 (25)	29 (30.5)	31 (21.4)	0.11

The data are medians and 25-75 percentiles, unless otherwise stated.

*Number of cells per microliter: the main lymphocyte subsets and terminal effector CD28^- ^CD8 cells were counted directly as absolute numbers per microliter of blood (single-platform flow cytometry).

**% of total lymphocytes. Naive and memory subsets were assessed as percentages of total blood lymphocytes, and their absolute counts were computed based on the absolute counts of CD4 and CD8 T cells

***The Immune Risk Phenotype (IRP) was defined as positive CMV serology plus at least one of the three following criteria: CD4/CD8 ratio < 1, CD8T-cells > 600 cells/μL, and CD28 negative T-cells > 300 cells/μL.

CMV serological status was determined in 246 patients and IRP status in 240 of these 246 patients. CMV serology was positive in 193 (78.5%) patients. In these CMV+ patients, we found immunological changes known to be associated with positive CMV serology (increased peripheral memory and effector CD4 and CD8 T-cells, data not shown). However, CMV status was not significantly associated with NI occurrence (*p *= 0.87). The rate of IRP+ patients was slightly higher in the NI+ group than in the NI- group, but the difference was not statistically significant (*p *= 0.11). The rate of nosocomial pneumonia was significantly higher in the IRP+ group (n = 60) than in the IRP- group (17/60, 28.3% versus 28/180, 15.6%; *p *= 0.036). Table 3 compares the IRP criteria in the 97 NI+ patients according to the initial site of infection. No significant differences were found across sites for a CD4/CD8 ratio < 1, CD8T-cells > 600/μL, CD28-negative T-cells > 300 cells/μL, or positive CMV serology. In-hospital mortality was 6% (n = 15/252) and did not differ between the IRP+ and IRP- groups (3/60, 5% versus 12/180, 6.6%, respectively; *p *= 0.76). However, in-hospital mortality was significantly higher in patients with nosocomial pneumonia than in the other patients (8/45,17.8% versus 7/207, 3.4%; *p *= 0.001).

**Table 3:** Immunological characteristics of the 97 elderly patients with NI according to the initial site of infection

	Lung n = 45	Urinary tract n = 33	Other sites n = 19	*p *value
IRP+	17(37.8%)	7(22.5%)	5(26.3%)	0.36

CD4/CD8 < 1	6(13.9%)	4(12.1%)	0 (0%)	0.26

CD8 T-cells > 600	9(20.9%)	4(12.1%)	4(21.5%)	0.60

CD28-CD8+ T-cells > 300	17(37.7%)	9(30%)	5(27.7%)	0.73

Positive CMV serology	39 (86.6%)	22 (68.7%)	15 (78.9%)	0.16

Patients with infection at several sites were classified based on the site involved initially.

## Discussion

In our elderly population, the immunological parameters associated with ageing of the immune system were more severely altered at admission in the patients who subsequently experienced one or more NIs than in those who remained free of NIs. More specifically, at baseline, patients with subsequent NIs had lower CD4/CD8 ratios, higher CD8 T cell counts, lower naive CD4 and CD8 T-cell counts, higher proportions of memory C8 T-cells, and higher counts of CD28^-^CD8 T-cells, compared to patients without NIs. CMV status was not associated with NIs. Moreover, patients exhibiting the IRP had a higher rate of nosocomial pneumonia than did the other patients.

To evaluate the immune system in our elderly patients, we looked at two sets of abnormalities associated with ageing: *(i) *classical immunology variables, which showed decreases in naive T-cell subsets and increases in memory/effector cells; *(ii) *and the IRP, a combination of immunological alterations specifically identified as associated with shorter survival in free-living healthy Swedes over 85 years of age [15-17]. In subsequent studies, such as the NONA study [19], the IRP was used in cohorts of individuals not selected for good health and was found to be unrelated to morbidity [20]. Both sets of abnormalities were present in our patients with NIs.

A limitation of our study is that only peripheral lymphocytes were counted. We did not evaluate other immune cells such as antigen-presenting cells or phagocytes, and we did not assess local factors at sites of infection.

In this study, we endeavoured to determine whether the risk of NI was predicted by immunological parameters. To our knowledge, the ability of immunological abnormalities reflecting immunosenescence to predict infections had not been studied previously in elderly in-patients. Wikby et al. first established that the IRP was a valuable predictor of mortality in very elderly people, a finding later confirmed in the NONA study [17,19]. However, the NONA study did not specify the causes of death and assessed the ability of the IRP to predict relatively long-term survival (6 years in patients aged 90 years or over). Interestingly, immunosenescence as assessed based on alterations in blood T-cell subsets has been described in Alzheimer's disease [21], and CMV infection is believed to contribute to the increased susceptibility of elderly individuals to many types of chronic antigenic stress such as cancer and autoimmunity [22].

Our data indicate a greater degree of immunosenescence in patients with NIs, compared to patients without NIs, despite the similar mean ages in the two groups. Moreover, patients with a positive CMV status and at least one immunological criterion for IRP were at increased risk for bacterial or viral pneumonia but not for bacterial infections of the urinary tract or other sites. This finding is consistent with the hypothesis that senescent CD8 T cells cannot respond properly to viral infections [10]

## Conclusion

The immunological alterations that characterise immunosenescence are more pronounced in elderly in-patients who subsequently experience NIs. The IRP profile is associated with nosocomial pneumonia in the relatively short term. The IRP parameters, which can be assessed easily on a routine basis and are known to predict higher mortality in elderly, have been shown by our study to also indicate a higher risk of NI, which is a clear manifestation of dysimmunity.

## Methods

### Patients and study design

We established a prospective cohort between July 2006 and November 2008 in a teaching hospital (approximately 1300 beds) in the Paris conurbation, France. Consecutive patients aged 70 years or over who were referred to the geriatric rehabilitation unit by acute medical or surgical units during the study period were eligible. Patients were included if they were medically stable at admission, required long-term care and rehabilitation, and had no terminal diseases (e.g., uncontrolled malignancy or severe dementia), fever, infection, cancer, or known dysimmunity. Patients were not included if they spent less than 72 hours in the rehabilitation unit. Study patients were followed up until discharge from the geriatric unit. The study complied with the Declaration of Helsinki and was approved by the Ile-de-France IX ethics committee (Paris, France). Written informed consent was obtained from each patient before study inclusion.

### Data collection

At study inclusion, a standardised questionnaire was used to collect age, gender, living conditions, and main reason for hospital admission. A blood sample was collected on the same day for counts of the main peripheral lymphocyte subsets (T, B, NK, naive T-cells, and memory/effector T cells) by flow cytometry and for CMV serology. Patients were monitored closely for NIs for 3 months or until discharge from the geriatric unit or death.

### Assessment of nosocomial infection

NIs were diagnosed by consensus between two investigators (CB and FT) who were unaware of the immunophenotype data. Once a week, these two investigators visited each hospitalised patient and reviewed the medical records with the attending physician and nurse. NI was defined as a well-documented infection that was neither present nor incubating at admission and that met the Centers for Disease Control definition of nosocomial infection [23]. NIs were diagnosed based on a combination of clinical findings (fever, pulmonary rales or dullness, dyspnoea, cough, purulent sputum, dysuria, urgency, suprapubic tenderness, clinical evidence of sepsis, and/or purulent drainage from a surgical incision), laboratory test results (blood and urine cultures, isolation of a pathogen from other specimens, and antigen- or antibody-detection tests), and findings from imaging studies (e.g., radiographs and computed tomography). We considered only active NIs, defined as under treatment with antimicrobial agents or considered on-going by the healthcare staff. When patients experienced more than one NI, only the first episode was considered in the statistical analysis. The study design did not involve specific tests for viral infections. We considered that nosocomial respiratory tract infections were either bacterial or viral and that infections at other sites were bacterial.

### Flow cytometry lymphocyte counts

At study inclusion, each patient had 4 ml of peripheral blood drawn and handled according to established clinical guidelines. Absolute counts of peripheral blood CD4 and CD8 T-cells, B cells, and NK cells were obtained directly using a Cyto-Stat tetraCHROME™ device (Beckman-Coulter, Hyaley, Florida). CD4 T cells were defined as CD3+CD4+ lymphocytes, CD8 T cells as CD3+CD8+ lymphocytes, NK cells as CD3- CD16+ and/or CD56+ lymphocytes, and B-cells as CD19+ lymphocytes. Reference values had been determined previously in our laboratory.

Naive, memory, and effector T cell counts can be determined based on the expression of CD45RA and CD62L [24]. Two additional combinations of monoclonal antibodies (from Beckman-Coulter, unless otherwise stated) were used to determine the relative percentages of CD8 and CD4 naive T cells (CD45RA^+^CD62L^+^), memory T cells (central, i.e., recirculating in secondary lymphoid organs, CD45RA^-^CD62L^+^; or peripheral, i.e., recirculating in effector sites, CD45RA^-^CD62L^-^), and terminal effector T-cells (CD45RA^+^CD62L^- ^or CD28^- ^for CD8 T cells) (CD45RA-CD62L) (BDPharmingen, Franklin Lakes, NJ)-CD3-CD4(Caltag)-CD8(Caltag) and CD28-CD3-CD8 (Caltag, Buckingham, UK)). The relative proportions of the naive and memory populations in peripheral blood vary with age [25].

### CMV serology

Serum from the blood sample taken at inclusion was used for the enzyme immunoassay Eti-CytoK-G Plus (Diasorin, Antony, France) to detect IgG antibodies to CMV.

### Immune Risk Phenotype criteria

To classify patients as IRP+ or IRP-, we defined the IRP [16] as positive CMV serology plus at least one of the following criteria: CD4/CD8 ratio < 1, CD8 T-cell count > 600/μL (the reference value used in our laboratory for routine testing), and CD28^-^CD8 T cell count above the threshold determined by Fagnoni et al. [9] (300/μL) in patients aged 80-99 years and > 210/μL in patients younger than 80 years.

### Statistical analysis

The prevalence of NI was computed using the total number of included patients as the denominator, and the 95% confidence interval (95%CI) was estimated. We compared the groups with and without NIs (NI+ and NI-, respectively) regarding baseline characteristics, clinical findings, and immunological findings. Since the counts of CD4 and CD8 T cells may vary independently from each other, the absolute counts are more reliable than the relative percentages. Immunological findings used in the comparison were the main lymphocyte subset counts, naive or memory/effector status of CD4 and CD8 T-cells, CMV serology, and IRP (as defined for our study). Qualitative variables were described as number (%) and compared using the Chi2-test or Fisher exact test, as appropriate. Quantitative variables were described as mean (± SD) or median (interquartile range, IQR) as appropriate and compared using the non-parametric Kruskal-Wallis test.

All tests were two-tailed and *p *values no greater than 0.05 were considered significant. No adjustments for multiple comparisons were performed. Data were analysed using STATA software SE11 (Stata, College Station, TX, USA).

## List of abbreviations

CMV: cytomegalovirus; Ig G: Immunoglobulin G; IQR: interquartile range; IRP: immune risk phenotype; NI: nosocomial infection; NK cell: natural killer cell.

## Competing interests

The authors declare that they have no competing interests.

## Authors' contributions

EP conceived and designed the study and is the guarantor for the study.

SBG contributed to the design, performed the statistical analysis, and interpreted the data.

AP performed the flow cytometry analysis and wrote the initial draft of the article, to which all the authors contributed subsequently.

FT, CB and KL collected the clinical data.

JPF participated in data interpretation and provided helpful comments about the manuscript.

All authors had full access to the study data and take responsibility for the integrity of the data and accuracy of the data analysis.

All authors read and approved the final manuscript.
